# Impact of sex on the utilization of defibrillation-capable cardiac implantable devices and outcome: results from the German device registry

**DOI:** 10.1007/s00392-025-02792-4

**Published:** 2025-12-01

**Authors:** Sorin Ștefan Popescu, Alessio Gasperetti, Johannes Brachmann, Lars Eckardt, Karl-Heinz Kuck, Stephan Willems, Patrick Lugenbiel, Ibrahim Akin, Christian Meyer, Claudia Schmidtke, Steffen Schneider, Matthias Hochadel, Jochen Senges, Roland Richard Tilz

**Affiliations:** 1 Department of Rhythmology, University Heart Centre Lübeck, University Hospital Schleswig-Holstein, Lübeck, Germany; 2 German Centre for Cardiovascular Research (DZHK), Partner Site, Hamburg Kiel,Lübeck, Lübeck, Germany; 3 Department of Cardiology, Johns Hopkins University, Baltimore, USA; 4 Centre Coburg GmbH II, Medical Clinic Cardiology, Angiology, Pulmonology, Coburg, Germany; 5 Department of Cardiology II – Electrophysiology, University Hospital Münster, Münster, Germany; 6 Department of Cardiology and Intensive Care Medicine, Asklepios Hospital St. Georg, Hamburg, Germany; 7 Department of Cardiology, Medical University Hospital , Heidelberg, Germany; 8 Medical Faculty Mannheim, University Medical Centre Mannheim, Heidelberg University, Mannheim, Germany; 9 Division of Cardiology, cNEP (Cardiac Neuro- and Electrophysiology Research Consortium), Teaching Hospital University of Düsseldorf, EVK Düsseldorf, Düsseldorf, Germany; 10 Foundation Institut Für Herzinfarktforschung, Ludwigshafen, Germany

**Keywords:** Sex medicine, Defibrillators, Implantable cardioverter–defibrillators, Cardiac resynchronisation therapy

## Abstract

**Background and aims:**

Implantable cardioverter defibrillators (ICDs) and cardiac resynchronization therapy with defibrillation-function (CRT-D) are widely used in patients with life-threatening arrhythmias or heart failure. We aim to investigate the impact of sex-specific differences on defibrillation-capable cardiac devices’ implantation and outcomes.

**Methods:**

The German DEVICE registry is a prospective, multicentre database of ICD and CRT device implantation. A total of 5330 patients receiving a defibrillation-capable device were prospectively enrolled in 44 centres between March 2007 and February 2014 and followed for 17 (13, 23) months.

**Results:**

A minority of patients in this registry was female 1017 (19.1%). The rate of CRT-D use among the defibrillator recipients was higher in women (32.4% vs. 28.0%; *p* = 0.006). The incidence of major periprocedural complications and in-hospital complications were higher in women (3.3% vs. 1.6%; *p* = 0.001 and 5.5% vs. 3.6%; *p* = 0.017, respectively). The 1-year all-cause mortality was 5.5% for women and 7.4% for men (*p* = 0.039), while the 1-year cardiovascular mortality was 4.1% and 6.2%, respectively (*p* = 0.012). Less women received device shocks during 1-year follow-up (10.7% vs. 13.8%; *p* = 0.023). Women receiving CRT-D had a lower non-device-related cardiovascular hospitalization rate than men.

System revision until discharge, in-hospital death and non-fatal complications during follow-up were comparable for men and women. Similar rates of all-cause and cardiac rehospitalizations were found.

**Conclusions:**

In this real-life patient cohort only a minority of patients was female. Female patients had a higher risk of major periprocedural complications and in-hospital complications but a lower all-cause and cardiovascular mortality. Less women experienced device-shocks during follow-up.

**Graphical Abstract:**

Overview of the results

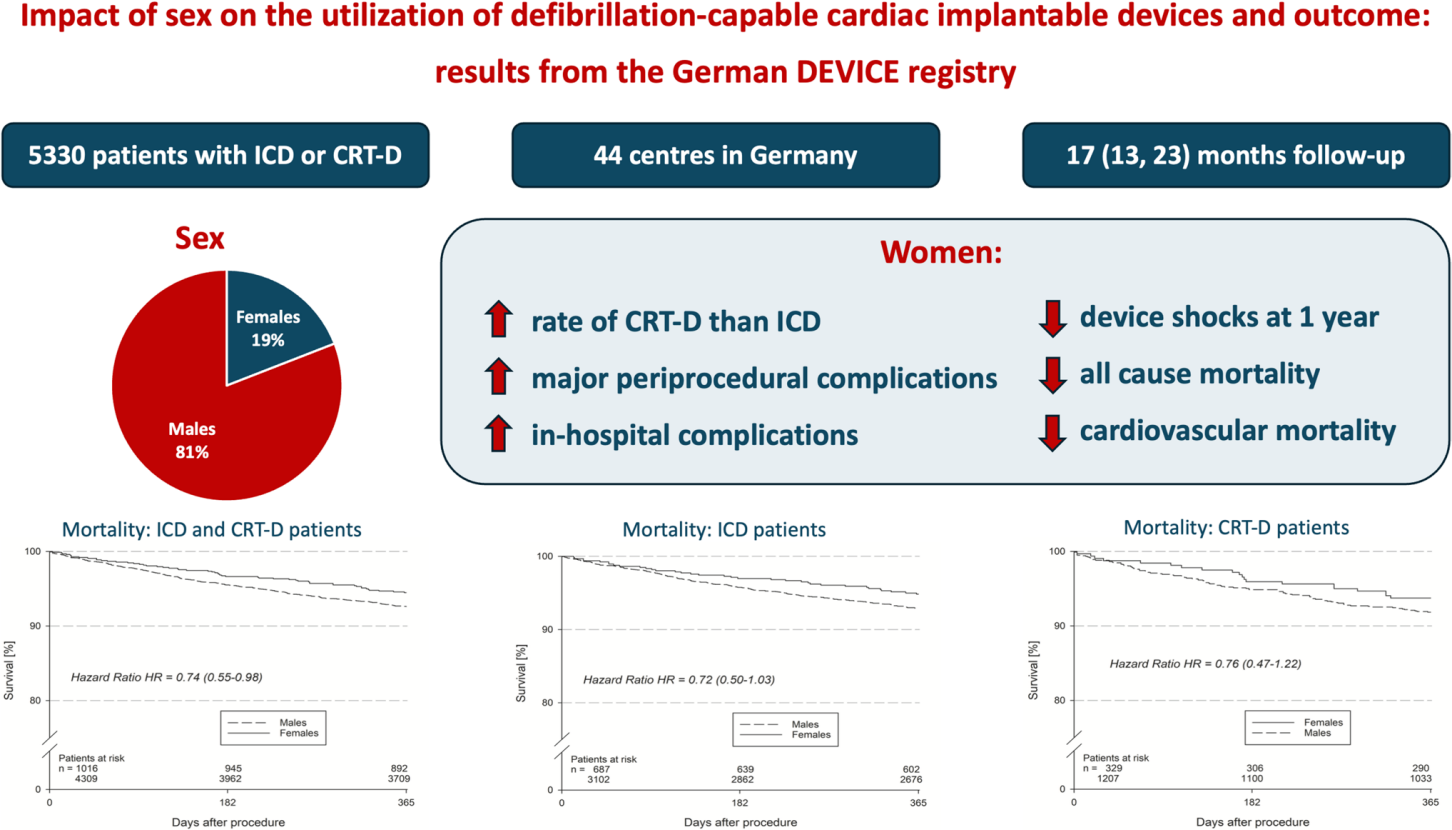

## Introduction

Randomized clinical trials have demonstrated the efficacy of implantable cardioverter defibrillators (ICDs) for both the primary and secondary prevention of sudden cardiac death (SCD) [1–7]. Current guidelines for the treatment of ventricular tachycardia (VT) and prevention of SCD recommend the use of ICD in appropriately selected patients with left ventricular ejection fraction (LVEF) ≤ 35% or a history of ventricular fibrillation (VF) or hemodynamically not tolerated VT [8].

In patients with heart failure (HF) with reduced LVEF (HFrEF), the use of cardiac resynchronization therapy (CRT) with defibrillation function (CRT-D) is superior to ICD alone for the reduction of morbidity and mortality [9–12]. Based on these findings, CRT is a class I recommendation in patients with symptomatic HF, with reduced LVEF (≤ 35%) and a left bundle branch block (LBBB) morphology QRS complex with a duration of ≥ 150 ms, despite optimal medical therapy, according to the latest guidelines [10].

The real-world data regarding the impact of sex on ICD and CRT prescription, efficacy and adverse events are scarce [13–16]. Despite important differences in baseline characteristics between male and female patients with implanted cardiac devices, several studies suggested a sex-based difference in device implantation and outcomes [16–18]. It has been demonstrated that female sex is independently associated with reduced implantation rates of any device [17, 19]. Moreover, when analysing the CRT devices recipients, women were less likely to receive a CRT-D than men [16, 18]. Among ICD recipients, several studies reported a higher incidence of both minor and major adverse events and complications in women as compared to men [20–23]. The rate of inappropriate therapies was significantly lower in females, both in CRT-D and ICD groups [12]. Most studies found no mortality difference between the sexes among the ICD recipients [21, 22]. When comparing the mortality in CRT recipients, the available data are conflicting. Most of the studies found a reduced mortality in CRT female patients as compared to men [14, 18, 24].

The German DEVICE Registry sought to investigate the impact of sex on defibrillation-capable cardiac devices implantation rate, adverse events and 1-year outcomes as seen in real-life circumstances. This registry presents one of the largest prospective observations of ICD and CRT-D patients with a particular focus on sex-specific differences.

## Methods

### Study population

The German Device Registry is a prospective, multicentre nationwide database including consecutive patients undergoing ICD or CRT-D device implantation or replacement between March 2007 and February 2014 [25, 26]. The registry was initiated by “Institut für Herzinfarktforschung” (IHF, Ludwigshafen, Germany) and included more than 70 parameters such as patients’ baseline characteristics, implant indication, device type, in-hospital complications and 1-year outcome. Forty-four centres enrolled patients in an internet-based, secure electronic database. Encrypted forms were transmitted to the IHF using a secure socket layer. All patients provided written informed consent.

This study was performed in line with the principles of the Declaration of Helsinki. Approval was granted by the Ethics Committee of Landesaerztekammer Rheinland-Pfalz.

For the current analysis, the patient population was divided into two sex-based groups.

### Follow-up

Data management and monitoring, including standardized follow-up calls were carried out by IHF. Patients were contacted by the device registry office 1 year after the device implantation or replacement and, using a standardized questionnaire, information about medical visits and hospitalization, changes in medication, subsequent cardiac procedures such as ablations and revascularization therapies, complications, arrhythmic events, syncope, and shocks, resuscitated cardiac arrests, myocardial infarction, working status and the patients’ satisfaction was obtained. Device interrogation was used to assess the arrhythmic events, and available medical records provided data on mortality.

### Statistical analysis

Categorical variables are presented as absolute and relative frequencies. They were compared using the Chi-square test or Fisher’s exact test, as appropriate. Continuous variables were reported as mean ± standard deviation (SD) when symmetrically distributed or for comparison with published data; otherwise, they were reported as median and interquartile range (1st quartile, 3rd quartile). Continuous variables were compared using the Mann Whitney-*U* test.

The Kaplan Meier method was used to estimate the 1-year mortality and 1-year incidence of a composite of death and shocks; the 1-year cardiovascular (CV) mortality was calculated by the Aalen-Johansen estimator considering non-cardiovascular mortality as a competing risk. The two groups were compared using the log-rank test. Moreover, multivariable Cox-regression models to assess the independent association between sex and 1-year overall and cardiovascular mortality were performed, adjusting for age, CAD, LVEF ≤ 35%, first implantation and device type (ICD-VVI, ICD-DDD, CRT-D). The results are reported as hazard ratios (HR) and 95% confidence intervals (CI). When performing the survival analyses, the unknown causes of death were counted as cardiovascular death.

A multivariable binary logistic regression to assess the independent predictors for in-hospital complications requiring medical intervention was conducted. The following variables considered clinically relevant were included in the multivariable model: sex, age, LVEF ≤ 30%, ICD-VVI, CRT-D, first implantation and secondary prevention implantation. The results are reported as odds ratios (OR) and 95% CI.

All *p*-values are two-sided and a *p*-value < 0.05 was considered significant. Data analysis was performed using the statistical analysis software SAS (release 9.4, SAS Institute, Inc., Cary, North Carolina. U.S.A.) and SPSS (version 29; IBM SPSS Statistics).

## Results

### Clinical presentation

Between March 2007 and February 2014, 50 centres enrolled 5330 patients receiving a defibrillation-capable device (ICD or CRT-D) in the German Device registry. Only a minority of them was female (1017, 19.1%). The two populations were similar in terms of age and body mass index (BMI) (Table 1). The female population was less likely to have coronary artery disease (CAD; 42.8% vs. 64.9%; *p* < 0.001), myocardial infarction (MI; 23.8% vs. 37.4%; *p* < 0.001), as well as a history of coronary revascularization (28.7% vs. 46.7%; *p* < 0.001). Females also had a shorter time between MI and device implantation/replacement (34 (6, 111) vs. 79 (6, 159) months; *p* = 0.031). Women were more likely to suffer from dilated cardiomyopathy (DCM; 38.0% vs. 31.9%; *p* < 0.001), hypertrophic cardiomyopathy (HCM; 4.3% vs. 2.9%; *p* = 0.024) or hypertensive cardiopathy (8.1% vs. 6.1%; *p* = 0.021). The incidence of primary electrical heart diseases was also higher in the female population (4.9% vs. 1.4%; *p* < 0.001), mainly driven by a higher incidence of long QT syndrome (LQTS) and arrhythmogenic right ventricular tachycardia (ARVC).

**Table 1:** Baseline characteristics

	Females 1017 (19.1%)	Males 4313 (80.9%)	*p* value	OR (95% CI)
**Age, years**	64.3 ± 14.7	65.4 ± 12.4	0.77	
**Age ≥ 65, % (*n*)**	59.1% (601/1017)	60.6% (2613/4311)	0.37	0.94 (0.82–1.08)
**BMI, kg/m**^**2**^	27.1 (23.0; 30.8)	26.9 (24.5; 30.0)	0.76	
• **CAD, % (*n*)**	42.8% (435/1017)	64.9% (2797/4313)	< 0.001	0.41 (0.35–0.47)
o **MI, % (*n*)**	23.8% (242/1017)	37.4% (1612/4313)	< 0.001	0.52 (0.45–0.61)
o **Time from MI, months**	34 (6; 111)	79 (6; 159)	0.031	
o **PCI, % (*n*)**	23.5% (59/251)	35.5% (345/973)	< 0.001	0.56 (0.41–0.77)
o **CABG, % (*n*)**	8.6% (87/1016)	19.1% (824/4313)	< 0.001	0.40 (0.31–0.50)
• **DCM, % (*n*)**	38.0% (386/1016)	31.9% (1377/4313)	< 0.001	1.31 (1.13–1.51)
• **HCM, % (*n*)**	4.3% (44/1016)	2.9% (127/4313)	0.024	1.49 (1.05–2.12)
• **Hypertensive cardiomyopathy, % (*n*)**	8.1% (82/1016)	6.1% (263/4313)	0.021	1.35 (1.04–1.75)
• **Valvular heart disease, % (*n*)**	1.9% (19/1017)	1.1% (46/4313)	0.036	1.77 (1.03–3.03)
• **Electrical heart disease, % (*n*)**	4.9% (50/1016)	1.4% (62/4313)	< 0.001	3.55 (2.43–5.18)
o **Brugada syndrome, % (*n*)**	0.5% (5/1016)	0.5% (20/4313)	0.91	1.06 (0.40–2.84)
o **Long QT syndrome, % (*n*)**	2.9% (29/1016)	0.4% (17/4313)	< 0.001	7.42 (4.06–13.57)
o **Short QT syndrome, % (*n*)**	0.0% (0/251)	0.0% (0/97)		
o **ARVC, % (*n*)**	0.8% (8/1016)	0.4% (16/4313)	0.075	2.13 (0.91–4.99)
**NYHA class**				
• **I, % (*n*)**	19.3% (166/860)	16.7% (667/3984)		
• **II, % (*n*)**	32.4% (279/860)	40.1% (1598/3984)		
• **III, % (*n*)**	45.1% (388/860)	40.1% (1596/3984)		
• **IV, % (*n*)**	3.1% (27/860)	3.1% (123/3984)		1.02 (0.67–1.55)
• **III/IV, % (*n*)**	48.3% (415/860)	43.1% (1719/3984)	0.006	1.23 (1.06–1.42)
**LVEF, % (*n*)**	34.2 ± 14.5	31.5 ± 12.0	< 0.001	
**LVEF ≥ 55%, % (*n*)**	13.8% (134/974)	6.7% (276/4147)	< 0.001	2.24 (1.80–2.79)
**LVEF < 35%, % (*n*)**	70.7% (676/956)	77.8% (3163/4067)	< 0.001	0.69 (0.59–0.81)
**Comorbidities**				
• **Stroke, % (*n*)**	3.6% (37/1016)	4.1% (178/4313)	0.48	0.88 (0.61–1.26)
• **PAD, % (*n*)**	2.1% (21/1016)	3.4% (147/4313)	0.028	0.60 (0.38–0.95)
• **Diabetes, % (*n*)**	27.0% (274/1016)	27.2% (1174/4313)	0.87	0.99 (0.85–1.15)
• **HBP, % (*n*)**	50.3% (511/1016)	53.1% (2289/4313)	0.11	0.89 (0.78–1.03)
• **COPD, % (*n*)**	3.5% (36/1016)	3.8% (163/4313)	0.72	0.94 (0.65–1.35)
• **CKD, % (*n*)**	14.9% (151/1016)	17.8% (769/4313)	0.024	0.80 (0.67–0.97)
o **Requiring dialysis, % (*n*)**	14.0% (7/50)	8.2% (17/208)	0.20	1.83 (0.71–4.68)

Values are counts, % (*n*), mean ± SD or median (first quartile, third quartile). OR = odds ratio, BMI = body mass index, CAD = coronary artery disease, MI = myocardial infarction, PCI = percutaneous coronary intervention, CABG = coronary artery bypass graft, DCM = dilated cardiomyopathy, HCM = hypertrophic cardiomyopathy, ARVC = arrhythmogenic right ventricular cardiomyopathy, NYHA = New York Heart Association, LVEF = left ventricular ejection fraction, PAD = peripheral artery disease, HBP = high blood pressure, COPD = chronic obstructive pulmonary disease, CKD = chronic kidney disease

Regarding left ventricular function, women had a higher mean LVEF (34.2 ± 14.5% vs. 31.5 ± 12.0; *p* < 0.001), were more likely to have a LVEF of > 55% (13.8% vs. 6.7%; *p* < 0.001) and less likely to have a LVEF of < 35% (70.7% vs. 77.8%; *p* < 0.001). The female population was more likely to have a New York Heart Association (NYHA) class of at least III (48.3% vs. 43.1%; *p* = 0.006).

No difference was seen with respect to history of stroke, diabetes mellitus, arterial hypertension, and chronic obstructive pulmonary disease (COPD), but the female population presented a lower prevalence of peripheral artery disease (PAD; 2.1% vs. 3.4%; *p* = 0.028) and chronic kidney disease (CKD; 14.9% vs. 17.8%; *p* = 0.024).

### Therapy indication

No difference between the sexes was seen in terms of implantation indication, with 59.5% of women and 59.4% of men receiving a defibrillator in primary prevention (*p* = 0.95) (Table 2). When analysing the secondary prevention indications, significantly more women had a history of VF (50.7% vs. 37.2%; *p* < 0.001) and significantly more men had a history of VT (47.5% vs. 34.5%; *p* < 0.001). Accordingly, females were more likely to have a history of resuscitation before implantation (54.9% vs. 45.2%; *p* < 0.001). Women also had a trend towards a higher prevalence of sudden death in their families (6.4% vs. 4.4%; *p* = 0.2). The MADIT-ICD VT/VF Score and MADIT-ICD non-arrhythmic mortality score were both significantly higher among males, with a more pronounced sex difference observed for the MADIT-ICD VT/VF score [27].

**Table 2:** Therapy indication

	Females 1017 (19.1%)	Males 4313 (80.9%)	*p *value	OR (95% CI)
**Type of prophylaxis**				
• **Primary, % (*n*)**	59.5% (605/1017)	59.4% (2561/4313)	0.95	1.00 (0.87–1.15)
• **Secondary, % (*n*)**	40.5% (412/1017)	40.6% (1752/4313)	0.95	1.00 (0.87–1.14)
**Arrhythmic history in secondary prevention**				
• **Ventricular fibrillation, % (*n*)**	50.7% (209/412)	37.2% (651/1752)	< 0.001	1.74 (1.40–2.16)
• **Ventricular tachycardia, % (*n*)**	34.5% (142/412)	47.5% (832/1752)	< 0.001	0.58 (0.46–0.73)
• **Syncope + inducible VF/VT, % (*n*)**	13.1% (54/412)	13.2% (231/1752)	0.97	0.99 (0.72–1.36)
• **Other arrhythmias, % (*n*)**	1.7% (7/412)	2.2% (38/1752)	0.55	0.78 (0.35–1.76)
**Clinical symptoms in secondary prevention**				
• **Resuscitation, % (*n*)**	54.9% (211/384)	45.2% (662/1466)	< 0.001	1.48 (1.18–1.86)
• **Syncope, % (*n*)**	22.1% (85/384)	25.0% (366/1466)	0.25	0.85 (0.65–1.12)
• **Presyncope, % (*n*)**	12.8% (49/384)	15.3% (225/1466)	0.20	0.81 (0.58–1.12)
• **Other symptoms, % (*n*)**	10.2% (39/384)	14.5% (213/1466)	0.026	0.66 (0.46–0.95)
**Familial history of SD, % (*n*)**	6.4% (16/251)	4.4% (43/968)	0.20	1.46 (0.81–2.65)
**MADIT-ICD VT/VF Score**	4 (3; 6)	7 (6; 8)	< 0.001	
• **Score ≥ 7, % (*n*)**	10.9% (48/439)	62.0% (1055/1701)	< 0.001	0.08 (0.05–0.10)
MADIT-ICD non-arrh. mort. Score*	2 (1; 4)	2 (1; 4)	0.018	
• **Score ≥ 3, % (*n*)**	44.1% (330/748)	48.5% (1667/3435)	0.029	0.84 (0.71–0.98)

Values are counts, % (*n*), mean ± SD or median (first quartile, third quartile). OR = odds ratio, VF = ventricular fibrillation, VT = ventricular tachycardia, SD = sudden death, * = without body mass index

### Procedural characteristics

A CRT-D device was implanted in 32.4% of women and 28.0% of men (*p* = 0.006). Significantly more men received an ICD-VVI device (51.8% vs. 46.2%; *p* = 0.001) (Table 3). The rate of first implantations (not revision) was significantly higher in the female population (88.9% vs. 85.3%; *p* = 0.003) as compared to men.

**Table 3:** Procedural data

	Females 1017 (19.1%)	Males 4313 (80.9%)	*p*value	OR (95% CI)
**Device type**				
• **CRT-D, % (*n*)**	32.4% (329/1017)	28.0% (1207/4313)	0.006	1.23 (1.06–1.43)
• **ICD (VVI), % (*n*)**	46.2% (470/1017)	51.8% (2233/4313)	0.001	0.80 (0.70–0.92)
• **ICD (DDD), % (*n*)**	21.4% (218/1017)	20.2% (873/4313)	0.40	1.08 (0.91–1.27)
**Device implantation, % (*n*)**	88.9% (901/1013)	85.3% (3672/4303)	0.003	1.38 (1.12–1.71)
**Device revision, % (*n*)**	11.1% (112/1013)	14.7% (631/4303)	0.003	0.72 (0.58–0.90)
**Rhythm at presentation**				
• **Sinus rhythm, % (*n*)**	81.0% (824/1017)	77.2% (3326/4307)	0.009	1.26 (1.06–1.50)
• **Atrial fibrillation, % (*n*)**	14.5% (147/1017)	19.6% (844/4307)	< 0.001	0.69 (0.57–0.84)
• **Pacemaker rhythm, % (*n*)**	5.2% (53/1017)	5.1% (221/4307)	0.92	1.02 (0.75–1.38)
• **Other rhythms, % (*n*)**	1.7% (17/1017)	1.8% (76/4307)	0.84	0.95 (0.56–1.61)
**Heart rate, bpm**	73.6 ± 15.3	72.5 ± 17.3	0.034	
**QRS duration, ms**	112 (95; 150)	118 (100; 150)	0.026	
• **QRS** ≥ **120 ms, % (*n*)**	47.9% (485/1013)	49.8% (2137/4292)	0.27	0.93 (0.81–1.06)
• **QRS** ≥ **150 ms, % (*n*)**	30.1% (305/1013)	27.5% (1180/4292)	0.095	1.14 (0.98–1.32)
**Duration of procedure, min**	62 (40; 105)	60 (41; 104)	0.92	
**Outpatient procedure, % (*n*)**	8.5% (86/1016)	9.8% (422/4305)	0.19	0.85 (0.67–1.08)
**Function test performed, % (*n*)**	75.8% (716/944)	74.0% (3010/4066)	0.25	1.10 (0.93–1.30)
• **Function test successful, % (*n*)**	98.9% (708/716)	99.5% (2996/3010)	0.041	0.41 (0.17–0.99)

Values are counts, % (*n*), mean ± SD or median (first quartile, third quartile). OR = odds ratio, CRT-D = cardiac resynchronization therapy with defibrillator capabilities, ICD = implantable cardioverter defibrillator, MR = magnetic resonance

The rhythm at presentation was sinus rhythm in 81.0% of women and 77.2% of men (*p* = 0.009) and atrial fibrillation (AF) in 14.5% of women and 19.6% of men (*p* < 0.001).

No difference was seen between the sexes in terms of elective and urgent implantation rates (*p* = 0.15) or procedure duration. The proportion of outpatient procedures was 8.5% for women and 9.8% for men (*p* = 0.19).

Female patients were more likely to develop in-hospital complications (5.5% vs. 3.6%; *p* = 0.017) (Table 4) as well as periprocedural complications requiring intervention (3.3% vs. 1.6%; *p* = 0.001). This difference was mainly driven by a higher incidence of pneumothorax (1.3% vs. 0.3%; *p* < 0.001) and haemothorax (0.4% vs. 0.0%; *p* = 0.014) in this group.

**Table 4:** In hospital adverse events/complications

	Females 1017 (19.1%)	Males 4313 (80.9%)	*p* value	OR (95% CI)
In hospital complications, % (*n*)*	5.5% (42/761)	3.6% (119/3318)	0.017	1.57 (1.09–2.25)
Device revision, % (*n*)*	2.3% (17/745)	2.2% (70/3213)	0.89	1.05 (0.61–1.79)
Myocardial infarction, % (*n*)*	0.0% (0/760)	0.0% (1/3318)	1.00	
Stroke, % (*n*)*	0.0% (0/760)	0.1% (2/3318)	1.00	
MACCE, % (*n*)*	0.1% (1/761)	0.4% (14/3329)	0.33	0.31 (0.04–2.37)
**Complications requiring intervention, % (*n*)**	3.3% (33/1013)	1.6% (69/4289)	0.001	2.06 (1.35–3.14)
• **Pericardial effusion, % (*n*)**	0.2% (2/1012)	0.1% (5/4289)	0.62	1.70 (0.33–8.76)
• **Hemothorax, % (*n*)**	0.4% (4/1012)	0.0% (2/4289)	0.014	8.51 (1.56–46.50)
• **Pneumothorax, % (*n*)**	1.3% (13/1013)	0.3% (12/4289)	< 0.001	4.63 (2.11–10.19)
• **Device-pocket hematoma, % (*n*)**	1.4% (14/1012)	1.2% (50/4289)	0.53	1.19 (0.65–2.16)
• **Cardio-pulmonary resuscitation, % (*n*)**	0.0% (0/252)	0.2% (2/971)	1.00	
• **Cardiogenic shock, % (*n*)**	0.0% (0/252)	0.0% (0/971)	1.00	
**Complications NOT requiring intervention, % (*n*)**				
• Pericardial effusion, % (*n*)*	0.3% 2/760	0.2% (6/3318)	0.65	1.46 (0.29–7.23)
• Hemothorax, % (*n*)*	0.0% (0/760)	0.1% (2/3318)	1.00	
• Pneumothorax, % (*n*)*	0.3% (2/761)	0.2% (7/3318)	0.68	1.25 (0.26–6.01)
• Local complications, % (*n*)*	0.6% (6/1012)	0.3% (15/4289)	0.27	1.70 (0.66–4.39)
• Other complications, % (*n*)*	1.2% (9/760)	1.2% (39/3318)	1.00	1.01 (0.49–2.09)
**Death, % (*n*)**	0.2% (2/1016)	0.3% (14/4310)	0.75	0.61 (0.14–2.67)
• **Survival time, days**	20 (17; 22)	12 (6; 16)	0.15	
• **Sudden/unexpected death, % (*n*)**	50.0% (1/2)	23.1% (3/13)	0.48	3.33 (0.16–70.91)
• **Cardiac death, % (*n*)**	50.0% (1/2)	57.1% (8/14)	1.00	0.75 (0.04–14.58)

Values are counts, % (*n*), or median (first quartile, third quartile). *P*-values are calculated using Fisher’s exact test. OR = odds ratio, MACCE = major adverse cardiac and cerebrovascular events (death, myocardial infarction, stroke). ^*^Data collected until March 2011 only

The multivariable binary logistic regression found the female sex as an independent predictor for in-hospital complications requiring intervention (OR: 2.07 (95% CI: 1.35–3.17); *p* = 0.0008), while the first device implantation was an independent predictor for a lower rate of such complications (OR: 0.55 (95% CI: 0.34–0.90); *p* = 0.017) (Table 5).

**Table 5:** Binary logistic regression for procedural complications requiring intervention

Variable	Adjusted OR (95% CI)	*p* value
Female sex	2.07 (1.35–3.17)	0.0008
Age (each 10 years)	1.09 (0.93–1.29)	0.30
LVEF ≤ 30%	0.70 (0.46–1.07)	0.099
ICD-VVI vs. ICD-DDD	0.61 (0.36–1.02)	0.058
CRT-D vs. ICD-DDD	1.16 (0.68–1.99)	0.58
First implantation	0.55 (0.34–0.90)	0.017
Secondary prevention	0.84 (0.53–1.32)	0.44

*OR *= odds ratio, *LVEF* = left ventricular ejection fraction, *ICD *= implantable cardioverter defibrillator, *CRT *= cardiac resynchronization therapy with defibrillation capabilities

The rate of complications not requiring intervention, the inhouse mortality rate as well as the number of device revisions until discharge was similar between both groups (Table 4).

### Follow-up

#### Survival

Median follow-up (FU) was 17.2 (13.2, 23.5) months for women and 16.9 (13.1, 22.9) months for men (*p* = 0.28). (Table 6).

**Table 6:** Follow-up data

	Females 1017 (19.1%)	Males 4313 (80.9%)	*p* value	OR (95% CI)
**FU available, % (*n*)**	96.9% (985/1017)	96.7% (4170/4313)	0.79	1.06 (0.72–1.56)
**Time to FU, months**	17.2 (13.2; 23.5)	16.9 (13.1; 22.9)	0.28	
**Death, % (*n*)**	10.0% (102/1016)	14.0% (602/4309)		
• **Cardiovascular, % (*n*)**	22.0% (22/100)	28.2% (159/564)		0.72 (0.43–1.19)
• **Non-cardiovascular, % (*n*)**	24.0% (24/100)	17.7% (100/564)		1.47 (0.88–2.43)
• **Unknown, % (*n*)**	54.0% (54/100)	54.1% (305/564)		1.00 (0.65–1.53)
• **Sudden/unexpected, % (*n*)**	23.8% (10/42)	24.2% (50/207)	0.96	0.98 (0.45–2.14)
• **Survival time, months**	10.9 (5.3; 28.6)	11.6 (4.4; 28.1)	0.92	
**MACCE, % (*n*)**	6.8% (67/985)	8.3% (348/4170)	0.11	0.80 (0.61–1.05)
**Non-fatal arrhythmic adverse events within first year in survivors**				
• **Resuscitation, % (*n*)**	0.9% (7/795)	0.4% (12/3292)	0.055	2.43 (0.95–6.19)
• **Defibrillator shocks, % (*n*)**	10.7% (81/759)	13.8% (440/3192)	0.023	0.75 (0.58–0.96)
• **Syncope, % (*n*)**	3.2% (17/532)	3.5% (75/2172)	0.77	0.92 (0.54–1.58)
• **Ventricular storm, % (*n*)**	1.5% (11/719)	1.5% (47/3047)	0.98	0.99 (0.51–1.92)
**Ablation, % (*n*)**	1.7% (6/362)	3.0% (43/1435)	0.16	0.55 (0.23–1.29)
**Non-fatal non-arrhythmic adverse events**				
• **Myocardial infarction, % (*n*)**	0.8% (6/707)	0.8% (23/2984)	0.83	1.10 (0.45–2.72)
• **Revascularization, % (*n*)**	2.0% (10/501)	2.7% (54/2011)	0.38	0.74 (0.37–1.46)
• **PCI, % (*n*)**	1.8% (9/501)	2.3% (46/2011)	0.50	0.78 (0.38–1.61)
• **CABG, % (*n*)**	0.2% (1/501)	0.4% (8/2011)	0.51	0.50 (0.06–4.01)
• **Stroke, % (*n*)**	1.0% (7/707)	1.0% (30/2981)	0.97	0.98 (0.43–2.25)
• Pulmonary embolism, % (*n*)*	0.0% (0/137)	0.5% (3/571)	0.40	
• Venous thrombosis, % (*n*)*	0.0% (0/137)	1.1% (6/571)	0.23	
**Device revision, % (*n*)**	9.9% (72/724)	8.2% (248/3040)	0.12	1.24 (0.94–1.64)
**Rehospitalization, % (*n*)**	43.3% (318/735)	41.1% (1273/3097)	0.29	1.09 (0.93–1.29)
**Device-related rehospitalization, % (*n*)**	16.0% (117/732)	14.2% (438/3085)	0.22	1.15 (0.92–1.44)
**Other CV rehospitalizations, % (*n*)**	10.9% (80/732)	11.2% (346/3085)	0.82	0.97 (0.75–1.26)

Values are counts, % (*n*), mean ± SD or median (first quartile, third quartile). OR = odds ratio, FU = follow-up, MACCE = major adverse cardiac and cerebrovascular events (death, myocardial infarction, stroke), PCI = percutaneous coronary intervention, CABG = coronary artery bypass graft, CV = cardiovascular. ^*^Data collected only from March 2011 until February 2014

**Fig. 1:**
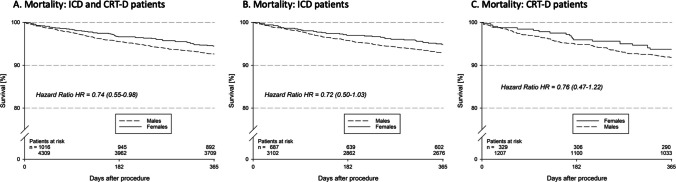
Kaplan–Meier estimated overall survival in the ICD and CRT-D population (**A**), ICDpopulation (**B**) and CRT-D population (**C**)

The Kaplan–Meier (KM) curves estimated a 1-year mortality of 5.5% for women and 7.4% for men (unadjusted HR 0.74 (0.55–0.99); *p* = 0.039) (Fig. 1). Interestingly, when analysing the ICD and CRT-D populations separately, the difference in mortality (5.2% for women and 7.1% for men; 6.3% for women and 8.2% for men, respectively) did not reach statistical significance (log rank *p* = 0.073 and *p* = 0.25, respectively).

The estimated incidence of death or defibrillator shock was 13.8% for the female population and 18.0% for the male population (*p* = 0.002). The cardiovascular mortality was 4.1% for women and 6.2% for men (HR 0.65 (0.47–0.91); *p* = 0.012).

In the multivariable regression, female sex was independently associated with a lower cardiovascular mortality (HR: 0.69 (95% CI: 0.50–0.97); *p* = 0.035), while there was only a trend towards a lower overall mortality in the female population (HR: 0.77 (95% CI: 0.58–1.04); *p* = 0.089).

#### Clinical follow-up and adverse events

Female patients had a higher incidence of resuscitation (0.9% vs 0.4%, *p* = 0.055), but a lower incidence of defibrillator shocks during the first year of FU (10.7% vs. 13.8%, *p* = 0.023) (Table 6).

No difference was observed between the groups in terms of incessant VT, non-fatal and non-arrhythmic adverse events, need of device revision or change in NYHA class during FU.

#### Rehospitalizations

The total number and device-related number of rehospitalizations after implantation was similar between both groups (43.3% for women vs. 41.1% for men; *p* = 0.29 and 16% vs. 14.2%; *p* = 0.82, respectively). Interestingly, in the CRT-D group, the rate of non-device-related cardiovascular hospitalizations was significantly lower among females (8.2% vs. 13.1%; *p* = 0.043), while the non-cardiovascular rehospitalizations rate was significantly higher in this population (22.8% vs. 17%; *p* = 0.041).

### Patients’ satisfaction

According to the results of the satisfaction questionnaire, 89.6% of women (*n* = 309/345) and 91.6% of men (*n* = 1202/1312) considered the treatment successful (*p* = 0.23), while only 1.4% of women and 1.9% of men rated the treatment as unsuccessful (*p* = 0.57). The perception of fear of receiving a device shock was significantly higher in the female population (20.4% vs. 9.7%; *p* < 0.001).

## Discussions

The present study is one of the largest prospective registries analysing the impact of sex on the implantation of defibrillator-capable devices and outcomes. The main findings are:

1. Only a minority of patients receiving ICD/CRT-D devices was female.
2. No difference was noted between the sexes in terms of primary and secondary prevention implantation.
3. Significantly more women with secondary prevention implantations had history of VF.
4. Among the patients receiving a defibrillator device, women were more likely to receive CRT-Ds, but less likely to receive ICD-VVIs.
5. Significantly more women developed major periprocedural complications and in-hospital complications.
6. The overall and cardiovascular mortality during FU were lower in the female population.
7. Women receiving CRT-D had a lower non-device-related CV hospitalization rate than men.
8. Significantly less women than men experienced device-shocks during FU.
9. The non-lethal, non-arrhythmic complications, medical visits and rehospitalizations during FU were similar between the sexes.

SCD is a leading cause of death worldwide, accounting for 40–50% of cardiovascular and 10–20% of all deaths in Europe [8, 28–31]. Incidence is consistently higher in men, even after adjustment for CAD, with an incidence rate ratio of 1.99 (95% CI 1.62–2.46) in those ≤ 75 years [30]. The MADIT II and SCD-HeFT trials showed that ICDs reduce all-cause mortality compared with medical therapy in patients with moderate heart failure [5, 6]. A meta-analysis of three secondary prevention trials demonstrated a 28% mortality reduction with ICDs versus medical therapy [32]. In selected patients, CRT-Ds were further shown to reduce mortality compared with ICDs alone [9, 11, 12].

### Study population

In our study population, only a minority of patients was female. These results are in line with other publications analysing the impact of sex on the ICD/CRT-D device implantation which reported a proportion of women among the implantable defibrillator implants recipients ranging from 10 to 35% [17, 19, 33–38]. A large publication aiming to determine the factors influencing the ICDs implantation among patients qualified for primary and secondary prevention treatment found the female sex as a negative predictor for defibrillator implantations in both patient cohorts [39]. Moreover, the same analysis showed that the sex disparities were persistent over years (1999–2005), despite a substantial increase in the rates of ICD use [39].

In their analysis, Curtis et al. suggested that the higher age of women at presentation might be one of the factors interfering with the implantation rate. However, after performing an age-stratified analysis, this hypothesis could not be confirmed [39]. Moreover, in our study, the age of women and men was similar. It is important to highlight that, even if the primary and secondary indications did not differ between sexes, significantly more women with a secondary prevention indication had a history of resuscitation and VF. This finding might suggest the need for a more severe event in order to consider the implantation among women. Another important finding of our study is that, although women presented with a higher LVEF at baseline, they were more symptomatic with respect to heart failure, as a larger proportion were classified as NYHA class III or higher. This observation is consistent with previous reports showing a greater symptom burden, poorer health status, and lower health-related quality of life in women with heart failure with preserved or reduced LVEF compared to men [40]. It further underscores sex-related differences in the clinical manifestation of heart failure and may be one of the factors influencing the selection of patients for ICD and CRT-D implantation.

In our study population, both the MADIT-ICD VT/VF score and the MADIT-ICD non-arrhythmic mortality score were higher among men, with a more pronounced difference observed for the MADIT-ICD VT/VF score: 62.0% of men compared with only 10.9% of women had a score of at least 7 points. This finding suggests a lower potential benefit of ICD implantation in women [27].

In spite of the striking difference in terms of female and male patients included in our registry, the design of the current analysis cannot evaluate the significance of this discordance, as it only includes ICD/CRT-D recipients.

### In hospital complications

In the present study, the in-hospital complications rate was higher in the female population, with a significantly higher incidence of major periprocedural complications in this group. It is important to note that the trend towards a higher complications rate in women did not reach the statistical significance level among patients receiving CRT-Ds only. These results are in line with other publications which reported a higher incidence of all ICD-implantation-related complications and adverse events among the female patients [20–23]. Russo et al. reported that women undergoing an ICD implantation had a higher incidence of pneumothorax requiring intervention, hematoma requiring evacuation and cardiac tamponade at 30 days postprocedural as compared to men [22]. In comparison, our results showed a higher incidence of pneumothorax among women in all treatment categories (ICD, CRT-D, all patients), but no difference in terms of hematoma and pericardial effusion requiring interventions. Moreover, a subanalysis of the MADIT-CRT trial demonstrated a higher incidence of major procedure-related adverse events, including pneumothorax, haemothorax and infections requiring interventions in the recipients of ICDs or CRT-Ds [41]. It is however important to note that in this subanalysis, no difference between sexes regarding the major procedure-related adverse events was seen in the ICD only group, but for the CRT-D only group and for the entire population [41]. The higher rate of pneumothorax among females might be at least partially explained by the smaller veins and thorax size in this population [13]. Thus, we underline the importance of the careful implantation technique and thorough monitoring, especially in the female population, in order to avoid these complications and limit their consequences. In the publication from Jamerson et al., the incidence of minor procedure-related adverse events was similar between sexes among the ICD and CRT-D recipients, findings also confirmed by our results [41].

### Mortality, hospitalization, arrhythmia recurrence and fear of receiving shocks

The MADIT-II trial showed no effect of sex on the survival of patients receiving an ICD in primary prevention [5]. Moreover, other publications showed a similar mortality between sexes among the ICD recipients [21, 22]. On the other hand, previous studies have proven that women with CRT-D criteria benefit more than men from this therapy in terms of all-cause mortality and heart failure hospitalization as compared to men [24, 36, 37].

Our analysis showed a significantly lower mortality for women than men in the overall population, but not in the ICD and CRT-D populations alone, where the trend towards a better survival of the female patients did not reach significance. Moreover, our results showed a lower non-device-related cardiovascular rehospitalization rate for women as compared to men in the CRT-D population, but not in the ICD population. These results might be explained by a lower HF-hospitalization rate for women receiving CRT-Ds in comparison to the men, as shown by de Waard et al. [36].

Maglia et al. used a propensity score-matched population to describe the sex-related differences in terms of first sustained ventricular arrhythmias (VA) events and device therapies (ICD and CRT-D) and showed that the female sex is significantly associated with a reduced risk of receiving any device therapy (HR: 0.59; *p* < 0.001) and device shocks (HR: 0.66; *p* = 0.021) during FU [34]. Although our registry does not provide details regarding the VA recurrence rate, the device shock rate was significantly lower in the female population. The literature results are controversial on this topic. While MacFadden et al. showed a significantly lower incidence of appropriate ICD shocks among women, other publications could only demonstrate a lower incidence of VA during FU among women receiving a CRT-D in primary prevention [21, 36].

Another important finding of the registry is that women reported a greater fear of receiving device shocks compared to men, consistent with previous studies which reported a greater concern regarding the ICD among women [42]. This psychological factor may contribute to the lower ICD and CRT-D implantation rates in women and should be addressed to improve both acceptance and quality of life. Effective strategies include patient education, counselling, psychological support, and device programming to reduce unnecessary shocks.

It must be emphasized that patients were enrolled between 2007 and 2014. During the past decade, clinical practice regarding device therapy has evolved substantially. Advances in heart failure management (e.g., introduction of SGLT2 inhibitors), broader implementation of guideline-directed medical therapy, refinement of device programming strategies, and improvements in implantation techniques may all have influenced patient outcomes [43, 44]. All these changes may limit the generalizability of our findings to contemporary practice, as their potential impact could not be assessed within the scope of the present study [8, 10, 44].

### Limitations

The current study represents a prospective, nationwide registry of the ICD and CRT-D recipients. Due to its nature, it comes with several limitations. First, the study includes only patients receiving a defibrillation-capable device and thus cannot assess the rate of device implantations between women and men. Second, due to the significant differences between sexes regarding the baseline characteristics, it is difficult to decide the real impact of the sex on different procedural and postprocedural findings. However, multivariable analyses were performed to compensate for different confounders. Third, when analysing the mortality, the unknown causes of death were counted as death from cardiovascular causes in both groups. Fourth, there was no information available regarding the rate of appropriate and inappropriate shocks. Fifth, not all data were available for each individual patient. Sixth, the median follow-up of 17 months is relatively short to fully evaluate long-term outcomes such as the incidence of ICD shocks and mortality. Nevertheless, the database still provides valuable insight into early and mid-term outcomes. Moreover, the database offers comprehensive information on indications, patient selection, procedural details, and complications, with sufficient follow-up to reliably assess postprocedural outcomes and sex-related differences.

## Conclusions

In this real-life patient cohort only a minority of patients was female. Female patients had a higher risk of major periprocedural complications and in-hospital complications but a lower all-cause and cardiovascular mortality in 1-year follow-up. Women receiving CRT-D had a lower non-device-related CV hospitalization rate than men. Significantly less women than men experienced device shocks during FU.

## Data Availability

Data supporting this study are curated at the Foundation Institut für Herzinfarktforschung, Ludwigshafen, Germany. Data are not shared openly but are available on reasonable request from the corresponding authors.
